# Assessing the impact of climate conditions on the distribution of mosquito species in Qatar

**DOI:** 10.3389/fpubh.2022.970694

**Published:** 2023-01-16

**Authors:** Furqan Tahir, Devendra Bansal, Atiq ur Rehman, Salah B. Ajjur, Sini Skariah, Samir B. Belhaouari, Hamad Al-Romaihi, Mohammed H. J. Al-Thani, Elmoubasher Farag, Ali A. Sultan, Sami G. Al-Ghamdi

**Affiliations:** ^1^Division of Sustainable Development, College of Science and Engineering, Hamad Bin Khalifa University, Qatar Foundation, Doha, Qatar; ^2^Department of Health Protection and Communicable Disease Control, Ministry of Public Health, Doha, Qatar; ^3^Division of Information and Computing Technology, College of Science and Engineering, Hamad Bin Khalifa University, Qatar Foundation, Doha, Qatar; ^4^Department of Electrical and Computer Engineering, Pak-Austria Fachhochschule Institute of Applied Sciences and Technology, Haripur, Pakistan; ^5^Department of Microbiology and Immunology, Weill Cornell Medicine-Qatar, Cornell University, Qatar Foundation-Education City, Doha, Qatar; ^6^Biological and Environmental Science and Engineering Division, King Abdullah University of Science and Technology, Thuwal, Saudi Arabia

**Keywords:** climate change, environment, malaria, mosquitoes, Naive Bayes, Qatar, vector-borne disease (VBD)

## Abstract

Qatar is a peninsular country with predominantly hot and humid weather, with 88% of the total population being immigrants. As such, it leaves the country liable to the introduction and dissemination of vector-borne diseases, in part due to the presence of native arthropod vectors. Qatar's weather is expected to become warmer with the changing climatic conditions across the globe. Environmental factors such as humidity and temperature contribute to the breeding and distribution of different types of mosquito species in a given region. If proper and timely precautions are not taken, a high rate of particular mosquito species can result in the transmission of various vector-borne diseases. In this study, we analyzed the environmental impact on the probability of occurrence of different mosquito species collected from several different sites in Qatar. The Naive Bayes model was used to calculate the posterior probability for various mosquito species. Further, the resulting Naive Bayes predictions were used to define the favorable environmental circumstances for identified mosquito species. The findings of this study will help in the planning and implementation of an active surveillance system and preventive measures to curb the spread of mosquitoes in Qatar.

## 1. Introduction

Mosquito-borne infections are a major health concern, with more than half of the world's population currently estimated to be at risk (1). World Health Organization (WHO) and the US Centers for Disease Control and Prevention (CDC), along with other international health agencies, have endeavored to improve surveillance for mosquito-borne diseases such as malaria and dengue fever, among others (2). Despite the rising global importance of mosquito-borne diseases, there are, to date no effective vaccines available to hamper the spread of these infections (3, 4). However, recently, a tetravalent chimeric vaccine, Dengvaxia (CYD-TDV), has been licensed for individuals 9–45 years of age in over ten dengue-endemic countries (5). Consequently, this puts vulnerable populations at increased risk of contracting mosquito-borne infections, thus affecting their quality of life. Several factors affect mosquito and mosquito-borne pathogens' life cycles and their geographical spread, including rapid population expansion and urbanization, animal migrations and trade, climate change, etc. (6). The impact of global climate change and environmental factors on vector-borne diseases (VBDs) can cause substantial changes in the transmission of VBD pathogens from vectors to the hosts (animal and human) (6).

Qatar is a small country with a total land area of 11,600 sq. km and a population of 3 million in 2022, and the majority (~88%) are immigrants and reside in the urban area (99%) (7). The climate in Qatar is hot and humid, and the average annual temperature is 27.1°C, with an average annual precipitation of 72 mm (8). The large immigrant force, urban area residents, and favorable weather conditions make Qatar susceptible to VBDs (9–11). Presently, as a non-endemic country, Qatar's vector control and surveillance initiatives are still inadequate (12). To address this problem, Qatar needs to strengthen its technical capacities in the field of entomology, with a particular focus on active surveillance and vector detection. Extensive long-term surveys are required to assess the effectiveness of mitigation strategies, which is a costly and time-demanding investment.

The presence of a single occurrence of VBD poses a health threat of transmission within different communities, especially those at higher risk of contracting the infection. Recently, our longitudinal and snapshot surveys in Qatar revealed that the southern house mosquito, *Culex quinquefasciatus*, is the most ubiquitous and populous mosquito species, followed by *Culex perexiguus*, suggesting a risk of West Nile virus (WNV) transmission. *Anopheles stephensi* was also widely distributed including in urbanized areas in Qatar, implying a possibility of local malaria transmission (9, 10, 12–14). In addition, *Aedes caspius*, a wetland mosquito, is also common, posing a risk of Rift Valley Virus (RFV) transmission.

Several studies have reported the effect of different factors, such as temperature, habitat, precipitation, migration, and relative humidity, on the mosquitoes' growth and abundance (15–18). Temperature is one of the essential abiotic factors responsible for insects' physiology, behavior, ecology, and survival (19). Mosquitoes adapt strategies to maintain body temperature and adapt to different climatic conditions by synthesizing heat shock proteins, thermoregulation, or changing their behavioral activity (20, 21). Moreover, temperature influences the length and duration of the extrinsic incubation period (EIP) (22). Relative humidity work in tandem with temperature to influence mosquito desiccation resistance (23), oviposition rate, adult mortality, adult survival, fecundity, hatching rates, sex proportion, and longevity ratio between female and male mosquitos (24). For example, mosquitoes have a shorter life span when relative humidity is <60%, but they live longer with increasing relative humidity (25). For *Aedes aegypti*, the gonotrophic cycle becomes shorter at higher mean temperatures and is optimal between 26 and 30°C (26). In addition, the temperature was the highest predictor of malaria transmission *via Anopheles* species, reaching its peaks at between 27 and 28°C (24, 27, 28). It was found that the risk of clinical malaria increased exponentially above 60% relative humidity and was twice as high at 80% (25). The *Culex* mosquito is the most prevalent mosquito species on the planet and a vector of various medically important viruses such as WNV, *St. Louis Encephalitis virus* (SLEV), and *Japanese Encephalitis virus* (JEV) (29–31). The *Culex quinquefasciatus* larvae also grow faster as the temperature rises, favoring the adult population (32–34).

Climate change is expected to get worse within the gulf region, which will result in higher dry and wet bulb temperatures, lower rainfalls, and increased humidity, among other weather changes (35, 36). Furthermore, a warmer climate means higher energy consumption due to the increased expulsion of waste energy from condenser/cooling towers as heat, thus further raising temperature and humidity (37–39). Additionally, a sudden increase in population during the FIFA World Cup 2022, coupled with favorable climatic conditions, is likely to facilitate the distribution and proliferation of mosquito species. Therefore, it is essential to conduct further studies that correlate the propagation of mosquito species and mosquito-borne diseases with environmental conditions.

Machine learning-based classification has become an increasingly valuable tool in recent years and can handle nonlinear, high-dimensional data with complex computable operations (40, 41). Classification algorithms, such as Naive Bayes (NB), neural networks (NN), decision tree (DT), Linear Discriminant Analysis (LDA), Support Vector Machine (SVM), and deep learning (DL) algorithms, are significant types of machine learning approaches (42–45). Due to its computational simplicity and efficiency, NB is consistently being enhanced and is frequently employed in various applications (46). For instance, Genoud et al. (47) implemented machine learning methods such as DT, NB, LDA, and SVM to predict the mosquitoes' species using optical signals. Their findings show that using optical sensors in conjunction with machine learning can be a feasible alternative or complementary to traditional mosquito population monitoring methods. Similarly, the present work focuses on implementing machine learning technique to compute the mosquitoes' specie probability distribution. In addition, the dependency of ambient conditions (ambient temperature and humidity) on the mosquito species distribution in Qatar is analyzed and discussed.

## 2. Materials and methods

### 2.1. Study area and environmental data

A total of nine different sites of diverse habitats were selected, as listed in Table 1, and the mosquitoes' samples were collected from August 2017 to August 2018, and details have previously been described elsewhere (13). The ambient conditions have been approximated using the data from six different stations of the Qatar Meteorological Department (QMD). The collected data is used to program the machine-learning algorithm, i.e., NB, to predict the probability distribution of certain mosquitoes species under environmental conditions.

**Table 1:** Site details for mosquitoes collection.

**Site #**	**Municipality**	**Habitat**	**Coordinates**	

			**Latitude**	**Longitude**
1.	Al Khor	Sewage basins	25.661767	51.517150
2.	Al Khor	Farm	25.760504	51.434899
3.	Al Rayyan	Farm	25.006483	51.194028
4.	Al Shahaniya	Zoo	25.439317	51.222233
5.	Al Shahaniya	Farm	25.171333	51.089283
6.	Al Shamal	Farm	25.959183	51.072083
7.	Umm Salal	Farm	25.466325	51.376284
8.	Doha	Widam company	25.236844	51.483064
9.	Al Rayyan	Farm	25.150400	51.363600

### 2.2. Sample collection and morphological characterization

To adjust for seasonal data, a set of repeated sampling sessions was conducted across the country to gather adult mosquito samples between August 2017 and August 2018. Farms, sewage basins, widam company and zoo were among the nine places chosen to represent various environmental sub-types that could influence mosquito breeding (Table 1). Over a thousand mosquitoes were collected and transported to the laboratory for morphological and molecular characterization, as reported previously elsewhere (13). Thousands of mosquitoes were collected, but the presence of considerable by-catches (attracted by the black light) and the poor quality of preservation did not allow all specimens to be properly sorted and identified. However, to obtain an estimate of sampling outcomes under our time constraints, we performed subsampling and analyzed one randomly chosen sample per month and per site. We analyzed 327 samples, yielding the detection of seven mosquito species or groups. The details are furnished in the previously published study (13).

### 2.3. Environmental conditions

**Figure 1:**
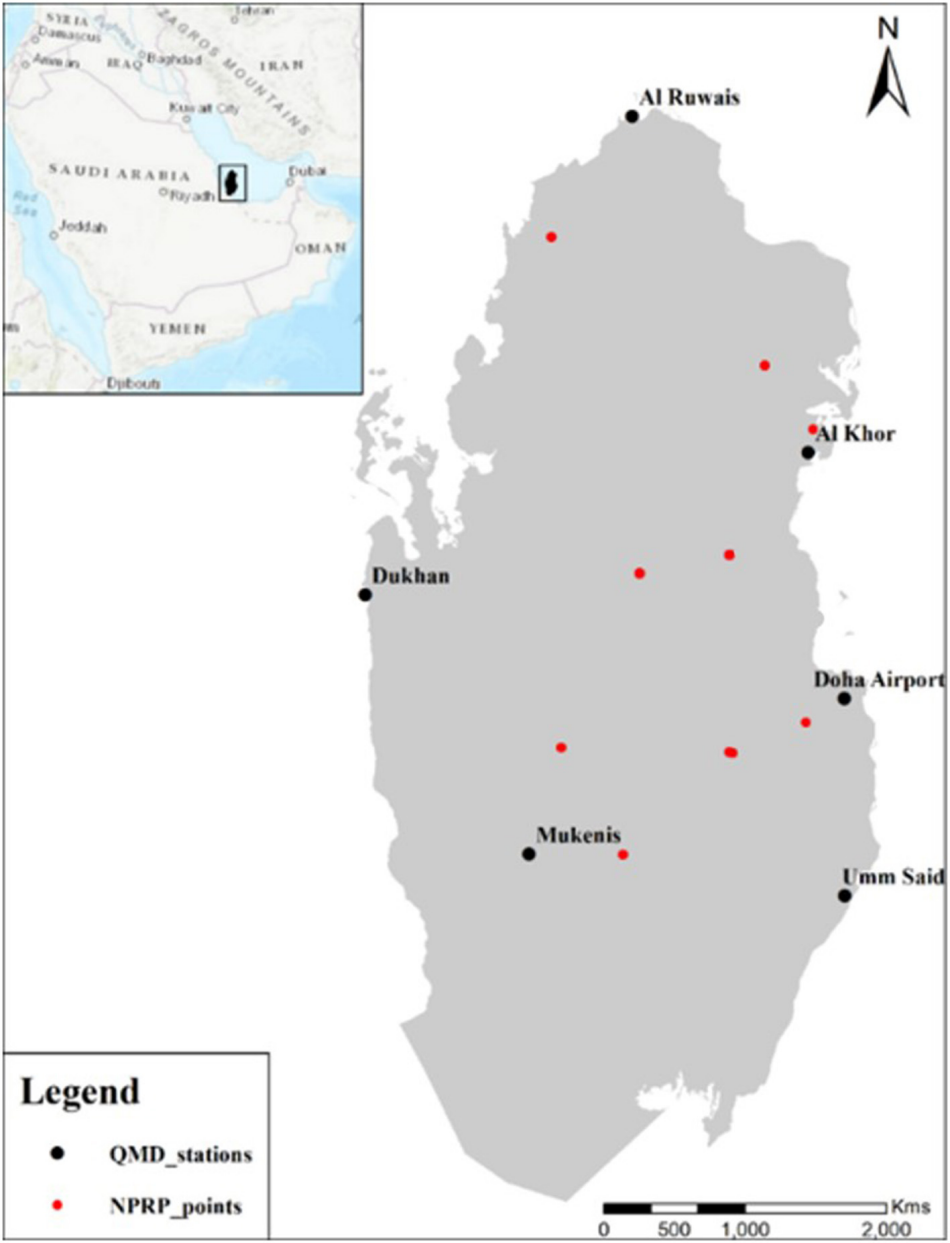
The location map, including Qatar Meteorological Department (QMD) stations in black and the studied nine sites in red color, respectively.

The dry-bulb (ambient) temperature and relative humidity observations from August 2017 to August 2018 were collected from the six meteorological stations of the Qatar Meteorological Department (QMD), Civil Aviation Authority. The locations of QMD stations are shown in Figure 1 in black dots. The Qatar Civil Aviation Authority stated that meteorological observations had undergone rigorous appropriate methodology to ensure homogeneity and quality. Furthermore, the six stations have had no systematic changes in their location or measurement techniques in the last three decades. The red dots represent the collection sites and habitats for mosquito collection. The temperature and humidity conditions of the nine sites considered in this work were derived from the data gathered from QMD stations. The inverse distance weighted (IDW) analysis tool within the geographic information system (GIS) was employed to estimate the weather conditions using the coordinates of sites (listed in Table 1) (48). The IDW is a widely used deterministic interpolation tool in the spatiotemporal characterization of climatic parameters. The IDW can be extended by reducing and extending methods to solve interpolation problems. It does not require any subjective assumption to select the semi-variogram model. Instead, it directly depends on the adjacent measured values or specific mathematical formulations for defining the smoothness of the surface. By definition, the IDW considers the measured points adjacent to the undetermined point, which is compared with the distant points. The general formula for the IDW is expressed as follows:


(1) $$\hat{Z}\;\left(s_{o}\right)=\;\sum {}_{i=1}^{N}\lambda_{i}\;Z\;(s_{i})$$

Where Z(s_i_) is the measured/determined value at the *i*th location; λ_i_ is the undetermined weight for the measured value at the *i*th location, which depends totally on the distance to the prediction location; s_0_ is the prediction location, and N is the number of measured values. For more information about the IDW tool, the readers can refer to (49).

### 2.4. Naïve Bayes (NB) algorithm

Since its creation, Naive Bayes (NB) has been effectively used in numerous applications. The Bayes' theorem (also known as Bayes' law or Bayes' rule), which carries Thomas Bayes' name, predicts the likelihood of an event based on knowledge of circumstances that may be associated with it in probability theory and statistics (50). Its ability to forecast future probabilities of events is quite effective. A family of straightforward “probabilistic classifiers” known as NB classifiers is based on using the Bayes theorem with strong (naive) independence assumptions between the features. NB is a conditional probability model that assigns probabilities to each of *C* possible outcomes or classes given a problem instance to be classified, represented by a vector *X* encoding some *n* attributes. Based on the observed data, it works by estimating the posterior probabilities of a given instance belonging to each conceivable class.

This study's goal was to forecast the existence of a certain mosquito species at a particular location under particular environmental circumstances, not to categorize various mosquito species. Therefore, in this study, instead of using the NB classifier, the posterior probabilities calculated by the NB were utilized to estimate the production of different mosquito species with variations in temperature and humidity in the state of Qatar. Due to its straightforward and easy implementation, the NB model has been chosen for this study, and previously this model has been used in several similar applications (47, 51).

Suppose a data *D* = {*X*^*i*^, *y*^*i*^}, where $X^{i}=\left(x_{1}^{i},x_{2}^{i},\ldots \;x_{n}^{i}\right)$ represents an instance with *n* features, *y*^*i*^ represents one of class *c* = 1, 2, …, *C* associated with an instance *X*^*i*^, and *i* = 1, 2, …*N* is the total *N* observations in *D*. For a new observation $\hat{X}$, NB assigns a label ŷ, which is predicted as:


(2) $$\begin{array}{l} ŷ={\text{arg max}}_{c\;=\;1,\;2,\;\ldots ,C\;}P\left(y=c|\hat{X}\right) \end{array}$$

where the posterior probability *P* is calculated by the Bayes theorem as:


(3) $$\begin{array}{l} ŷ={\text{arg max}}_{c\;=\;1,\;2,\;\ldots ,C\;}\frac{P\left(y=c\right)P\left(\hat{x_{1}},\hat{x_{2}},\;\ldots \ldots ..,\hat{x_{n}}\;|y=c\right)}{P\left(\hat{X}\right)} \end{array}$$

Based on the features independence supposition, the joint probability is expressed as:


(4) $$P(y=c|\hat{x_{1}},\hat{x_{2}},\;\ldots \ldots ..,\hat{x_{n}}\;)\propto P(y=c)\prod {}_{i=1}^{n}P(\hat{x_{i}}|y=c\;)$$

This indicates that the conditional distribution over the class variable *c* under the aforesaid independence assumptions is as follows:


(5) $$P\left(y=c|\hat{x_{1}},\hat{x_{2}},\;\ldots \ldots ..,\hat{x_{n}}\;\right)=\frac{1}{Z}P(y=c)\prod {}_{i=1}^{n}P(\hat{x_{i}}|y=c\;)$$

where $Z=P\left(\hat{X}\right)=\underset{c}{\sum }P\left(y=c\right)P\left(\hat{X}|y=c\right)$ is a scaling contact depending only on the feature variables $\hat{x_{1}},\hat{x_{2}},\;\ldots \ldots ..,\hat{x_{n}}$ which are known.

The NB classifier calculates the posterior probabilities as defined in Equation (5) and classifies an observation as belonging to one of the classes as defined in Equation (2). In this study, the posterior probabilities calculated by the NB were utilized to estimate the production of different mosquito species with variations in temperature and humidity in the state of Qatar. Table 2 enlists the details of the arguments for the NB algorithm. Furthermore, the numerical experimentation was carried out in MATLAB R2019 on a 2.9 GHz 6-Core Intel Core i9 Mac-Book Pro.

**Table 2:** Arguments details for NB algorithm.

**Arguments**	**Description**
*D*	Dataset
*X*	Observations in D
*Y*	Known Class labels of X
*N*	The number of observations in D
*C*	The number of classes
*C*	The label of an observation
*N*	The dimensions of X

## 3. Results and discussion

### 3.1. Morphological identification and characterization of vector species

Among the various species of mosquitoes identified, the most abundant were *Culex quinquefasciatus* and *Cx. (Cux.) perexiguus*, (which are often difficult to distinguish from dried—and frequently damaged—adults), found at all sites. The only *Anopheles* species found was (*Cellia*) *stephensi* at seven sites (excluding site 1: Al Khor—Sewage basins and site 6: Al Shamal—Farm). Moreover, *Culex quinquefasciatus* was prevalent throughout the year, while *An. stephensi* was only moderately abundant between October and November and then between June and July. *Aedes (Ochlerotatus) caspius*, was observed at six different sites and at various times throughout the year but was found in small numbers. Our entomological survey collected samples from farms, garden centers, industrial regions, sewage lakes and treatment plants, urban building areas, wetlands, worker housing, and a zoo to account for diverse elements affecting mosquito reproductive capabilities and distributions.

**Figure 2:**
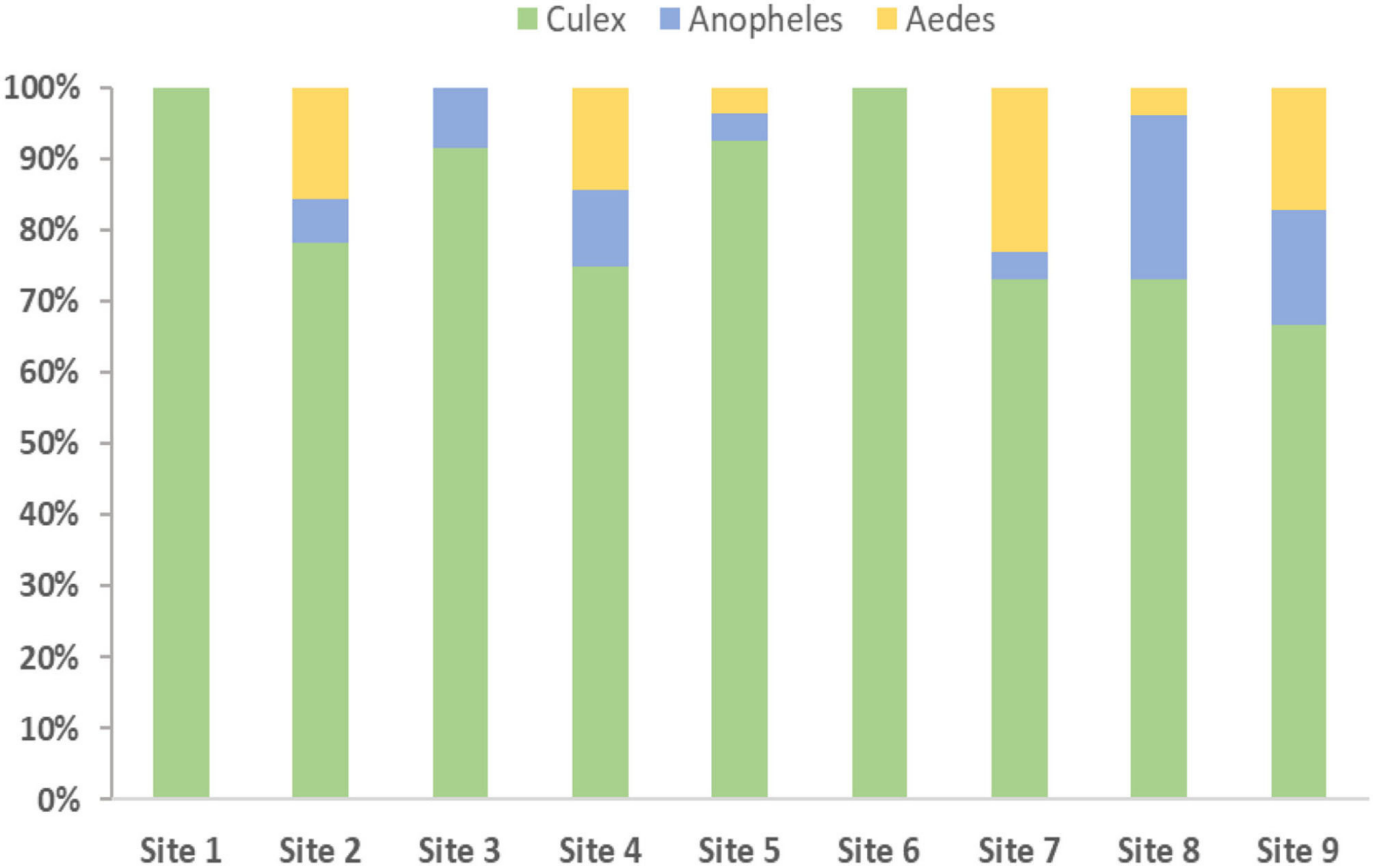
Mosquitoes' species collection and identification for different sites.

Furthermore, every inspected area contained one or more mosquito species, with the southern house mosquito species *Culex quinquefasciatus* having the most widespread geographical range due to the high degree of adaptability to their environment. Additionally, our findings were consistent with the species' known preferences and posed a risk to public health through disease transmission (52). For generalization, the mosquitoes were grouped into *Culex, Anopheles*, and *Aedes*, as shown in Figure 2. Most of the mosquitoes' samples were collected from Doha and Al-Rayyan municipalities. This data was used in combination with the weather dataset to predict the mosquitoes' probability distribution. To appropriately assess the risk of mosquito-borne diseases, routine field monitoring and analyses are required to address research gaps in terms of breeding, distribution, and biting inclinations of various mosquito species presently found in Qatar.

### 3.2. Environmental conditions

**Figure 3:**
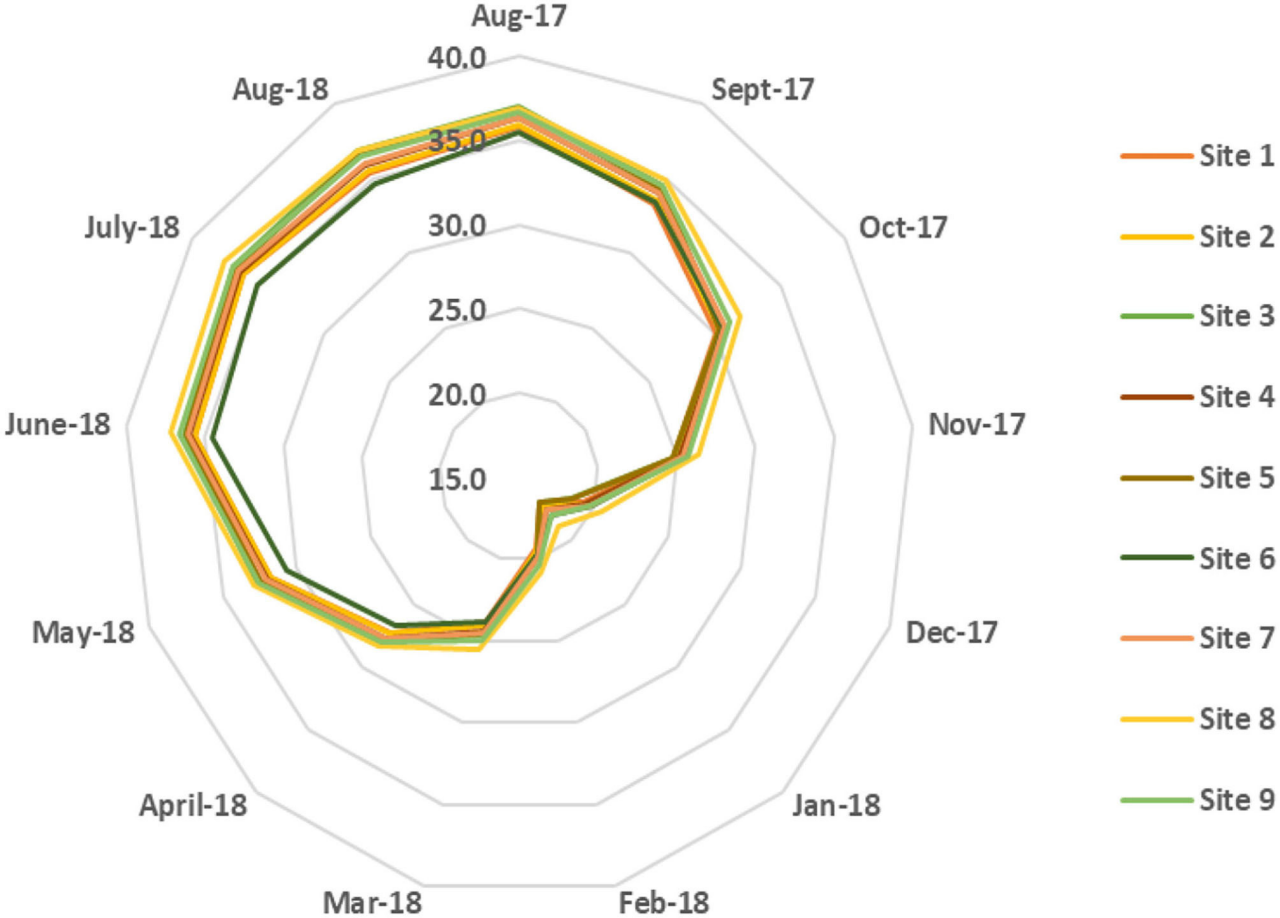
Monthly average temperature distribution in [°C] for selected sites.

Figure 3 exhibits the monthly average ambient temperature and relative humidity distributions for selected sites from August 2017 to August 2018. The lowest monthly average temperature recorded in January ranged from 16.9 to 18.8°C. The temperatures steadily rose as the summer months approached. For the period from June to August, the monthly average temperature reached 35 to 37°C. However, the maximum temperature during summer can potentially reach well beyond 45°C.

**Figure 4:**
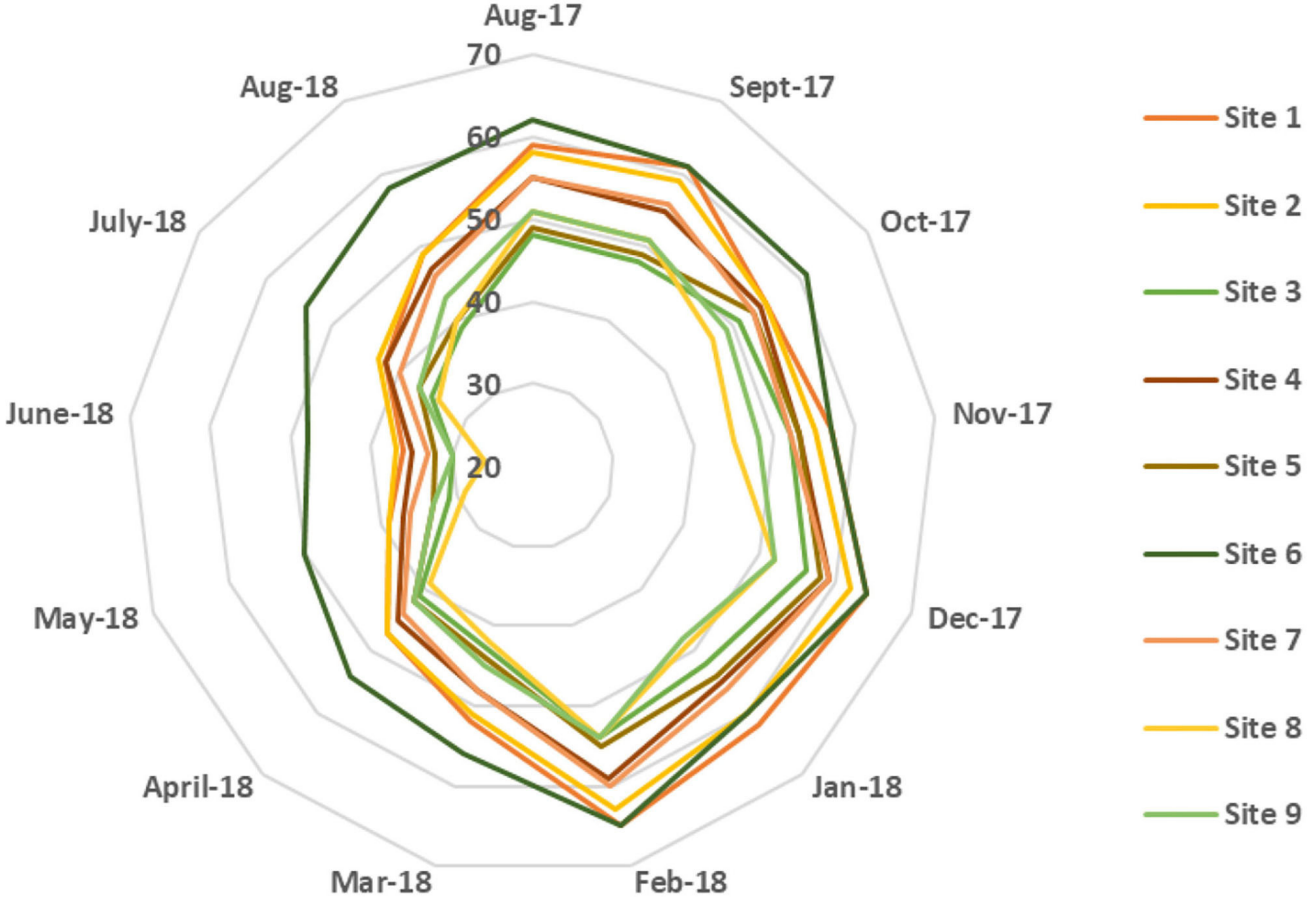
Monthly average relative humidity distribution for selected sites.

Moreover, the monthly average temperature variation was small between different sites, and the maximum variation in temperature was <2°C. The monthly average temperature at site 8 was found to be slightly higher than at the other sites. The monthly average relative humidity variation for different sites is shown in Figure 4. The humidity index in Qatar typically tends to be high, averaging in the range of 45–65% during the half of summer (August and September), fall, and most of the winter season until the following February. Afterward, the average monthly humidity decreased to the lowest at 26% (June) and then increased again during the summer, thus continuing the cycle. In contrast with the temperature profile, the variation of relative humidity (12–22%) among different sites was statistically significant. In summary, site 8 was relatively warmer and less humid, and site 6 exhibited lower monthly average temperature and higher relative humidity than other sites, respectively.

### 3.3. Probability distribution

**Figure 5:**
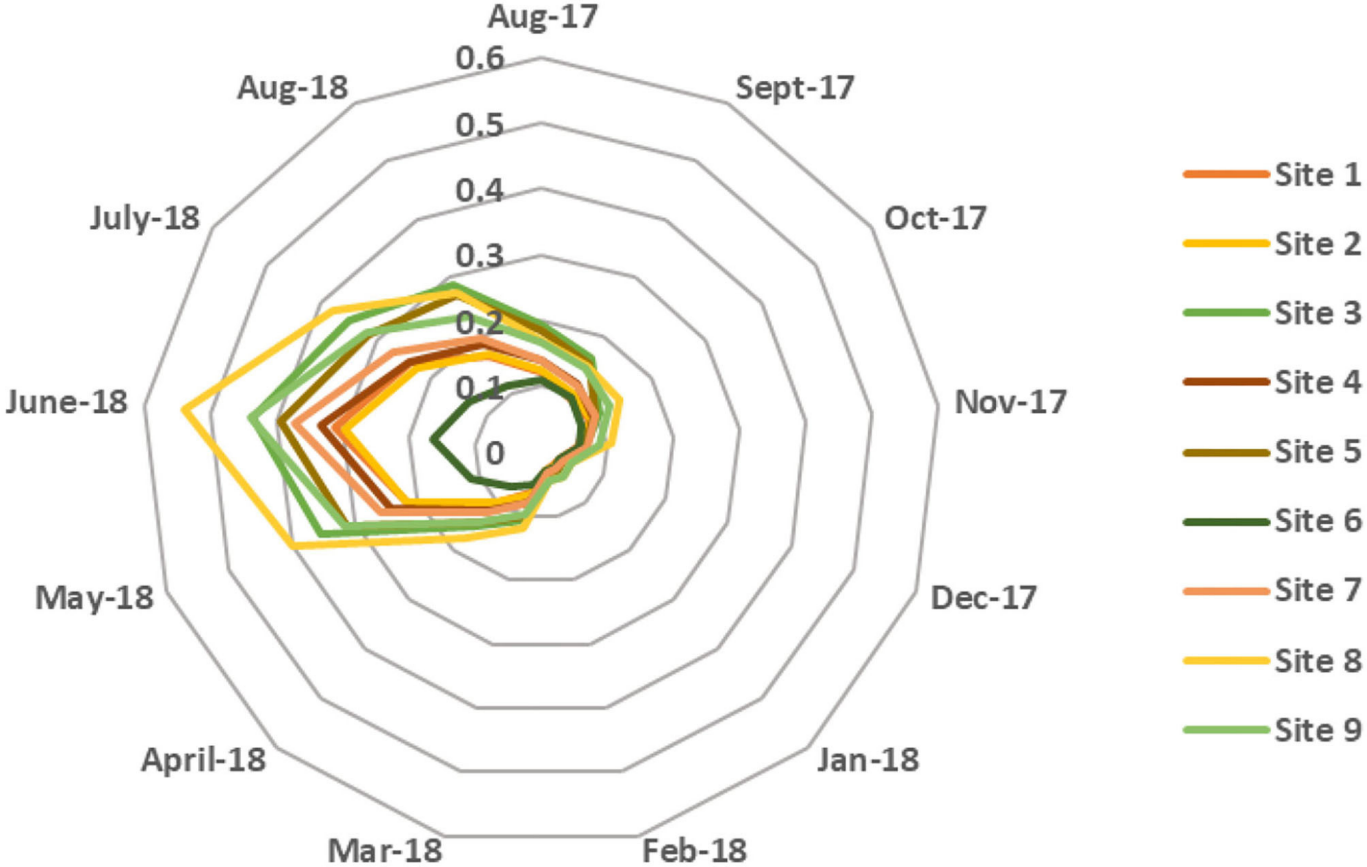
Monthly average probability distribution of *Anopheles* mosquito for different sites.

**Figure 6:**
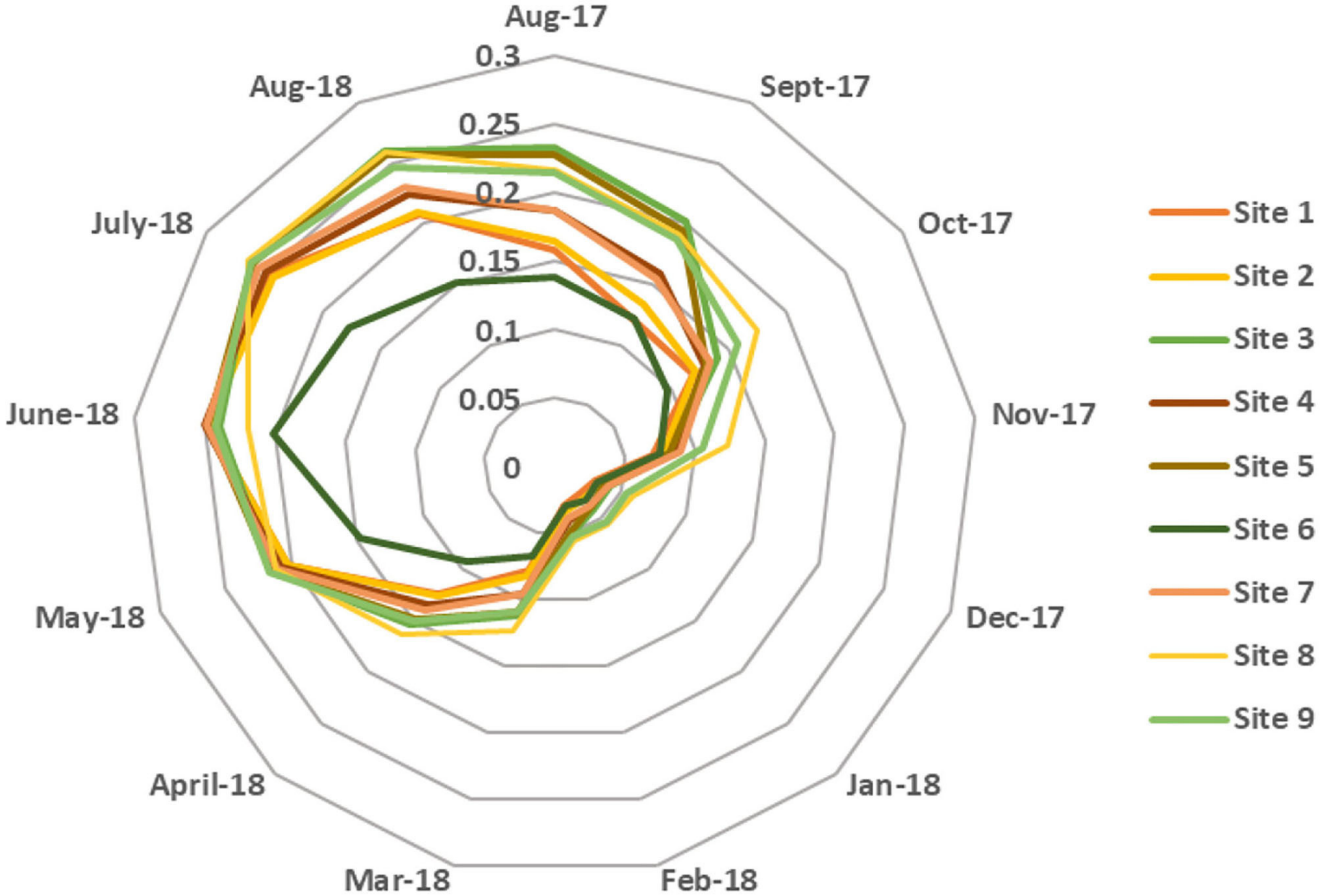
Monthly average probability distribution of *Aedes* mosquito for different sites.

At first, the accuracy of the NB model is evaluated by conducting a 10-fold cross-validation, and the observed model's accuracy was found to be 81.34%. The model's accuracy can be further improved by improving the class imbalance problem of the collected dataset. As, many samples in this dataset belong to *Culex* class only, therefore the classifier learning can be biased toward this particular class. *Culex* families of mosquitoes were found to be more abundant in Qatar; hence, the probabilities were computed only for *Anopheles* and *Aedes* mosquitoes. The results presented in sections Morphological identification and characterization of vector species and Environmental conditions were used to train the NB algorithm to predict the distribution of mosquito species. Figure 5 shows the probability distribution of *Anopheles* (malaria vector) throughout the year for the different collection sites. It was observed that the probability of the presence of *Anopheles* mosquitoes is lowest during the winter season (≤ 0.05) and increases during summer, reaching its peak in June, given that they thrive in warmer temperatures. However, it varies when compared between different sites as the probability of *Anopheles* mosquito at site 6 is just 0.162 and at site 8 is 0.538. This significant difference could be due to site 8 (Doha—Widam Company) being much warmer and less humid than site 6 (Al Shamal—Farm), which has lower ambient temperature and higher humidity compared to the other sites. Figure 6 represents the *Aedes* mosquito probability distribution from August 2017 to August 2018 for the different collection sites. It follows a similar trend to that of *Anopheles* mosquito distribution, i.e., lower presence in the winter (December to February) and higher in the summer season, respectively. However, in the summer season, the probability of presence of *Aedes* mosquitoes is lower than that of *Anopheles* mosquitoes. Like *Anopheles* mosquito probability distribution, the probability for *Aedes* mosquito tends to decrease at higher humidity. Thus, temperatures around 35°C with humidity levels of 35–45 % are suitable environmental conditions for the abundance of both species, i.e., *Anopheles* and *Aedes*.

These conditions, compounded by a largely immigrant population of Qatar who travels to and from VBD endemic countries (9), have the potential to further propagate *Anopheles* and *Aedes* species of mosquitoes.

**Figure 7:**
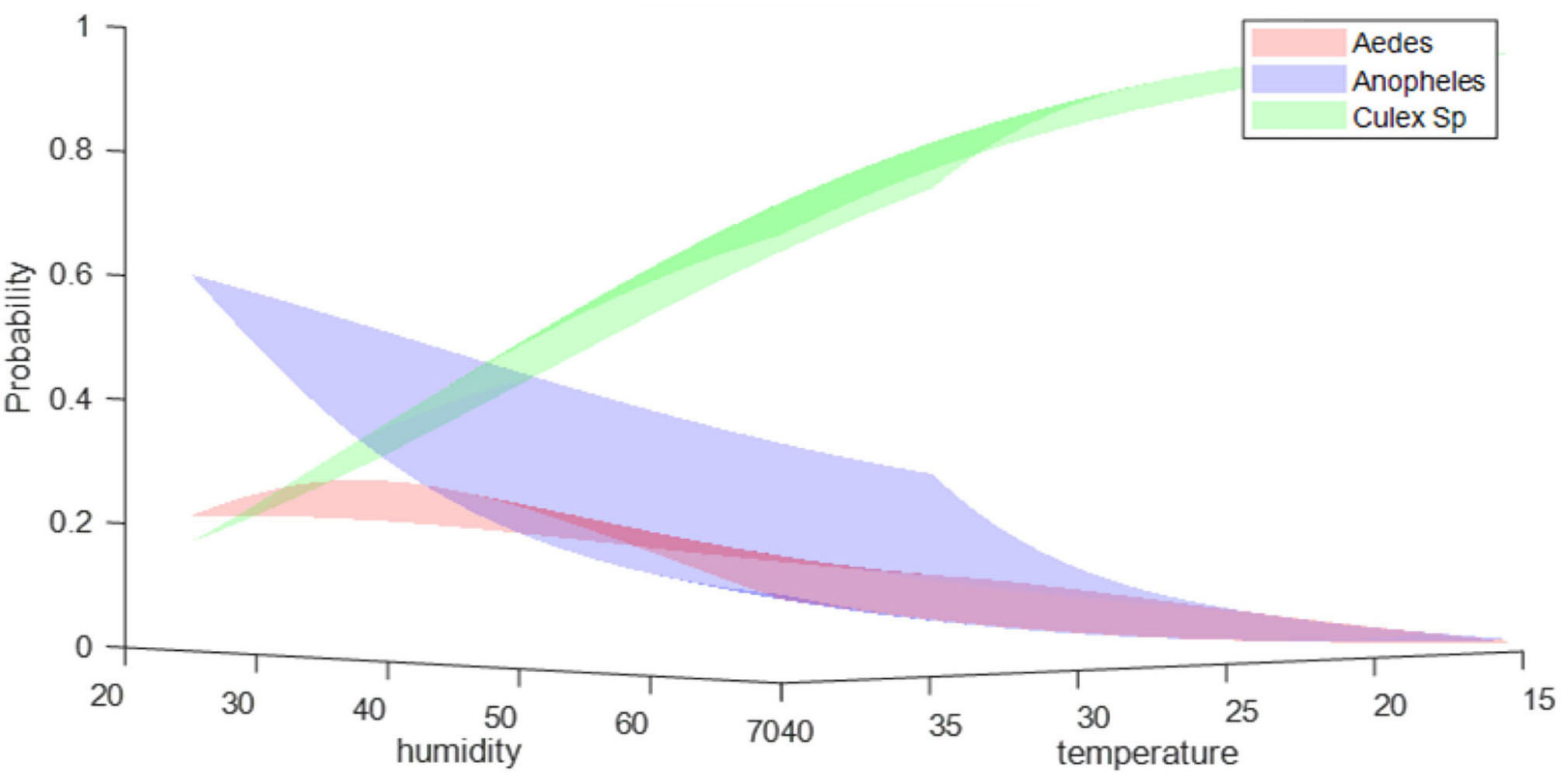
Predicted probability distribution of mosquito species with respect to environmental conditions (ambient temperature and humidity).

Figure 7 shows the comparative distribution probability of mosquitoes with respect to temperature and humidity. For both species (*Anopheles* and *Aedes*), the likelihood increased at higher temperatures and lower humidity. However, for *Aedes* mosquito, the probability increased with the declining humidity levels to a certain point before it started to decrease again. The suitable environment for *Aedes* mosquitoes was observed to be in the range of 35–40°C temperature and 35–45 % relative humidity. Furthermore, our findings align with the studies (28, 53, 54). Although machine-learning models are good at predicting the mosquitoes' specie probability distribution, the only drawback these models have is the requirement of labeled data for model learning. It is often difficult to collect and label different mosquito species, especially the species those are rarely found at specific sites. Therefore, it might not be feasible to apply such models in scenarios where the labeled data is not available. However, if the labeled data is available to train machine-learning models, these models can easily predict the presence of certain species at certain sites in advance, and therefore, precautionary measures could be taken in advance to control the production of mosquitos on those sites, if necessary.

## 4. Conclusion and recommendations

Climate change affects the environment and humans, and also has social and economic implications. The increased greenhouse gas emissions on the part of human activities contribute to warming temperatures on Earth. This will, in turn, engender further dissemination of infectious diseases *via* vectors of concern and floods—all of which are threats to human health. Such events propel human beings beyond the limits to which they can adapt; therefore, death rates increase. Depending on the area that a population is living in, the relationship between temperature and mortality varies. The changes in temperature and other climate variables control how vectors are redistributed. Such changes contribute to the burden of VBDs and the deterioration of human health.

This study conducted mosquitoes species identification and propagation at nine different sites of various habitats in Qatar using the Naive Bayes algorithm. It was found that the *Culex* mosquitoes are the most abundant, followed by the *Anopheles* and *Aedes* types. The *Anopheles* and *Aedes* types were observed at seven and six collection sites, respectively. Results show that higher temperatures and lesser humidity enhance the chance of both species (*Anopheles* and *Aedes*). However, the risk of an *Aedes* mosquito increases as the humidity decreases, but only to a certain point and then drops. Temperatures between 35 and 40°C and relative humidity levels between 35 and 45 % are ideal for *Aedes* mosquitoes. Our results reaffirm the need for a robust surveillance system working in combination with the environmental sector and an extensive, multivariate dataset to provide a clearer outlook of the potential distribution and abundance of mosquito species.

A resilient healthcare structure to protect against mosquito-borne infection requires (1) continuous monitoring and surveillance and (2) a large dataset to provide a clearer picture of the likelihood of mosquito species abundance and distribution. In addition, there is an increased risk of VBD transmission during FIFA World Cup 2022 as Qatar prepares to host the event, with over a million attendees expected to arrive in the country. Therefore, policy development and implementation are vital for adequate emergency preparedness and response for VBDs.

## Data availability statement

The raw data supporting the conclusions of this article will be made available by the authors, without undue reservation.

## Author contributions

Conceptualization: FT, DB, AR, and SA-G. Methodology: FT, DB, AR, and SB. Software: FT, SA, and AR. Formal analysis: FT and DB. Investigation: DB, HA-R, and MA-T. Resources: EF, AS, and SA-G. Data curation and visualization: FT and AR. Writing—original draft preparation: FT, DB, and AR. Writing—review and editing: SS, AS, EF, and SA-G. Supervision: SA-G, AS, and EF. Project administration: AS and SA-G. Funding acquisition: SA-G, AS, EF, and DB. All authors contributed to the article and approved the submitted version.
